# Effect of Resource Spatial Correlation and Hunter-Fisher-Gatherer Mobility on Social Cooperation in Tierra del Fuego

**DOI:** 10.1371/journal.pone.0121888

**Published:** 2015-04-08

**Authors:** José Ignacio Santos, María Pereda, Débora Zurro, Myrian Álvarez, Jorge Caro, José Manuel Galán, Ivan Briz i Godino

**Affiliations:** 1 INSISOC, Universidad de Burgos, Escuela Politécnica Superior, Edif. “La Milanera”, Burgos, Spain; 2 CaSEs, Department of Archaeology and Anthropology, IMF-CSIC, Barcelona, Spain; 3 CONICET-CADIC, Bernardo Houssay, 200, Ushuaia, Argentina; 4 CaSEs, GSADI, Department of Sociology, Universitat Autònoma de Barcelona, Barcelona, Spain; 5 BSC-CNS, Barcelona Supercomputing Center, Nexus I. C/ Gran Capità, 2–4, Barcelona, Spain; 6 Department of Archaeology, University of York, York, United Kingdom; Universidad Carlos III de Madrid, SPAIN

## Abstract

This article presents an agent-based model designed to explore the development of cooperation in hunter-fisher-gatherer societies that face a dilemma of sharing an unpredictable resource that is randomly distributed in space. The model is a stylised abstraction of the Yamana society, which inhabited the channels and islands of the southernmost part of Tierra del Fuego (Argentina-Chile). According to ethnographic sources, the Yamana developed cooperative behaviour supported by an indirect reciprocity mechanism: whenever someone found an extraordinary confluence of resources, such as a beached whale, they would use smoke signals to announce their find, bringing people together to share food and exchange different types of social capital. The model provides insight on how the spatial concentration of beachings and agents’ movements in the space can influence cooperation. We conclude that the emergence of informal and dynamic communities that operate as a vigilance network preserves cooperation and makes defection very costly.

## Introduction

Discovering when cooperative practices first emerged, how they evolved and what factors brought them into existence and influenced their continuity is an issue of paramount importance for the social sciences [1–8]. Social and environmental interaction, innovation and conflict shape social evolution, producing a high degree of variability. This variability provides an interesting and promising field for identifying the rules and mechanisms embedded in the emergence, maintenance and change of social cooperation.

In the case of hunter-gatherer societies, two challenges must be faced. First, the history of these societies is mostly traced by studying their material culture and cooperative practices are difficult to identify in the archaeological record. Second, these societies are attractive laboratory cases for attempting to understand, in small-scale contexts, what kinds of situations, behaviours and attitudes fuel or prevent social cooperation.

Using ethnoarchaeology as our methodological framework [9–14], we performed a series of experiments using agent-based model computer simulation to tackle cooperative practices in a hunter-gatherer context [12]. Ethnoarchaeology is understood here not only under the perspective of “living archaeology” [15,16], but as a methodological approach in archaeology that aims to develop new methods and hypotheses [9–11].

The main objective of the case study is to assess the development of cooperation in a hunter-fisher-gatherer society that called itself “Yamana” during the 19^th^ and 20^th^ centuries [17] and inhabited the southernmost part of the Fuegian archipelago (South America). The WWHW model (Wave When Hale Whale) is based on data provided by the rich ethnographic record about this society and focuses on a particular set of practices, norms and decisions that arose whenever a cetacean was stranded on the coasts of Yamana territory. According to written sources, when Yamana people discovered a beached whale they could either announce it publicly through four smoke signals and share it with others, or keep all its resources for themselves [17–20]. When the people signalled their find, an aggregation event could take place where a high number of families that usually experienced their everyday lives in small groups would gather together to take advantage of the natural accumulation of resources. This unpredictable but regular event (see below) fostered youth initiation ceremonies and strengthened social bonds and norms. Within the Yamana these kind of cooperative attitudes were encouraged not only through education, but also by means of social regulations [17]). Promotion occurred through mechanisms such as reputation, but so did different types of punishment [17,21,22]

In the first stage of our research, we established the main entities, variables and scales of the model and explored the effects of specific parameters in promoting cooperation, such as social reputation, the chance to find the resource and, consequently, to detect a defector (measured though *vision* parameter) [12]. The results showed the high relevance of social reputation and imitation strategies for maintaining cooperative practices even with low visibility values (since people agents can only replicate observable behaviours).

This paper focuses on a key organisational element in hunter-gatherer societies: mobility strategies and the distribution of resources. In our previous article, human agents moved randomly and whale agents appeared from time to time on different coastal places or “patches”. In this experiment, Lévy flight movement has been incorporated into the model in order to reproduce more realistic behaviour for people agents. Previous studies have shown that this kind of movement represents an important mobility pattern for hunter-gatherers when searching for resources that are heterogeneously distributed [23].

At the same time, the information provided by different researches showed that although cetacean strandings are random phenomena, they tend to reoccur in the same geographical areas. To build a more realistic environment, in this paper we define areas with differential probabilities for whales to become stranded.

Therefore, these experiments allow us to define the real possible scenarios that could help to raise cooperative behaviours within the context of aggregation events, considering the geographical setting and these groups’ management of the territory.

## Materials and Methods

### Archaeological and ethnographic sources

#### Mobility and cooperation

It is widely accepted that mobility strategies play an important role in structuring hunter-gatherer organisation and how they manage inhabited territories [24]. There are two central questions in relation to this topic: the reasons why hunter-gatherers move around the landscape in a particular way and what their movement patterns are like. Thus, knowledge and predictability about resource distribution, as well as food preferences, play an essential role in mobility strategies.

The well-known forager-collector continuum, proposed by Binford in 1980 and strongly based on resource distribution, has been one of the more prominent models applied to tackle this issue [25]. According to Binford, foragers make residential moves in pursuit of resources while collectors acquire more distant resources, sending small logistic groups out to collect and bring them back to a central camp. However, several researchers have pointed out that mobility was not simply linked to resource depletion but also strengthened social ties, helped in the search for mates and also facilitated the exchange of information and goods (for example [26–30]).

Hunter-gatherer displacement patterns were traditionally explained as random walks like in Brownian motion, a concept originally formulated to define the movement of microscopic particles. Nowadays, various models and approaches seek to understand the underlying mechanisms that lead to a particular movement pattern [31]. One such model is the Lévy flight pattern, which has been observed in many animal species such as wandering albatrosses [32], spider monkeys and marine predators [33], although some of them have been recently proven to contain flaws [31,34]. Moreover, the theoretical work of Viswanathan et al. [35] states that Lévy flight with exponent μ = 2 is an optimal search strategy in environments with scarce, randomly placed resources that can be revisited because they are not depleted during consumption. This has led to the emergence of the Lévy flight foraging hypothesis, later confirmed by empirical studies (e.g. [36]). This foraging strategy is deemed optimal, and thus central in human evolution [33]. The Lévy flight pattern has been observed not only in human and animal mobility, but also in online games [37] and in human cognition [38].

Lévy flight has also been applied to the study of hunter-gatherer mobility, with an exponent near the optimum value to explain the movement pattern of the Dobe Ju/’hoansi living in deserted areas of Botswana and Namibia [39], whose seasonal behaviour is driven by water availability. Other empirical research found that approximately half the foraging patterns of the Hadza societies in northern Tanzania match Lévy walk patterns, showing that more than one foraging pattern can coexist [23].

#### Movement of coastal hunter-fisher-gatherers: the Yamana case study

Yamana people were aquatic hunter-fisher-gatherers (following [40]) specialised in the management and exploitation of marine resources who used canoes to move across the territory [17]. Their diet was mostly based on the consumption of sea mammals, seashells, birds, guanacos and fish. These resources seem to have had a relatively homogeneous spatial distribution and most of them were not seasonally constrained [21]. Historical documents show that the Yamana had high residential mobility with frequent and short movements, similar to a foraging strategy according to Binford’s model.

Written sources point out that people self-identified in relation to specific spaces where they were born or lived [19], naming them, for example *Canagush Yamana*, *Putroaya Yamana*, *Wullaia Yamana* or *Lashuf Yamana* [17,41,42] as “Yamana” is the word for “Humanity” in their own language [43]. These places included bays and beaches stretching several kilometres. However, longer distances between residential locations were also recorded [20,44].

The Yamana usually moved in very small groups, but on some occasions several social units or households could spend some time together (visiting relatives or performing social activities during aggregation events [21]). Following ethnographical sources, aggregation events could happen when cetaceans or fishes were stranded on the coasts, providing a natural and abundant source of food. These specific aggregations afforded the scenario for a rise in cooperative practices to the extent that people who discovered a whale drifted ashore had to notify the nearby families or groups using smoke signals in order to share the abundance of food and raw materials [17,20]. Breaking this rule brought social sanction and conflict among the Yamana people [45].

Three interesting points were recorded in historical documents in relation to Yamana mobility patterns and aggregation events. First, several accounts hold that those episodes brought together “local people” as well as families that came from different places [46]. Second, the news of a beached whale spread from distant areas [47,48]. Third, some accounts mention that the Yamana made specific trips along their territory in order to detect stranded whales [49].

Under the WWHW model, mobility played an important role since it allowed Yamana people to discover not only beached whales, but also non-cooperative agents. Lévy flight walks may be useful to model Yamana mobility since, in the case of cetaceans, we are dealing with a resource spread across space [23,50].

Current research on cetacean strandings has showed that they do not occur homogeneously, but tend to concentrate geographically in relation to migratory and reproductive routes. Malvinas-Falklands and Tierra del Fuego Islands are in fact one of the 23 most frequent areas worldwide for *Ziphiidae* (beaked whale) strandings [51]. Ethnographic and historical information from Tierra del Fuego, combined with present-day records, provide a partial record of these phenomena and enable us to identify areas where strandings occur more frequently [52].

Mobility strategies related to strandings would probably have changed throughout the years in relation to the higher frequency of a particular species. Whale strandings have mainly been recorded between March and May, although different sources give contrasting information. In fact, records from the late 19^th^ century indicate a concentration of strandings between March and April [53].

Therefore, according to historical and ethnographical information, there would have been areas and periods where and when the possibility of a cetacean getting stranded would have been higher. Although this fact is not considered under our model, Yamana people would probably move within the territory taking into consideration the heterogeneous distribution in time and space of this particular and valuable resource.

### An agent-based model

The next sections describe the model following the ODD documentation protocol [54]. The computational model is implemented in NetLogo 5.0 [55] and the corresponding source code may be downloaded at the following website http://www.openabm.org/model/4249.

#### Overview: purpose

The Wave When Hale Wale (WWHW) [12] is an agent-based model designed to allow the exploration of the emergence, resilience and evolution of cooperative behaviours in hunter-fisher-gatherer societies, using the Yamana society with an example when confronted with a dilemma of whether to share resources.

In this extension of the model, we test the influence of some factors that might affect the evolution of cooperation:

- A mechanism of indirect reciprocity to promote cooperation that conditions people’s capacity to gain social capital from others in aggregations (as in [12]).
- The characteristics of natural events that generate cooperation opportunities, i.e. stochasticity, unpredictability, spatial distribution and limited visibility.
- Human walking patterns, in particular random walk and Lévy flight movements.

We also suppose an evolutionary mechanism of imitation of the two strategies (i.e. always cooperate and always defect) considered in the model.

#### Overview: entities, state variables, and scales

There are two kinds of agents in the model: people and whales. People agents represent households/canoes moving around the environment looking for a beached whale. A whale agent is an unpredictable and scarce resource, which implies a valuable and perishable food resource for people. From time to time, a whale beaches and any people agent that finds it needs to make a decision about whether to call other people to share the resource or not. People are mobile agents while whales are static. The number of people in the model remains constant during simulation.

**Fig 1:**
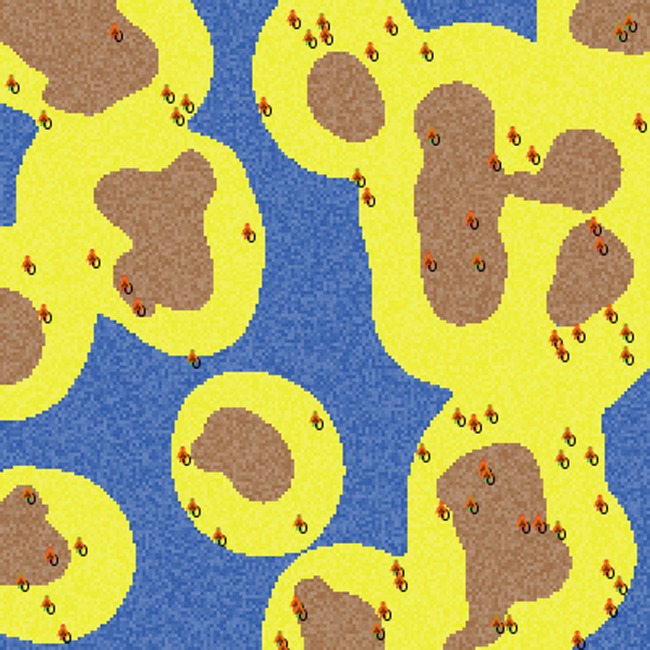
Snapshot of a 201x201 patch environment. Blue cells represent water, yellow represent beach and brown stands for land.

The environment is defined by a square grid of MxM cells, i.e. patches. Patches can represent beach, water or land (Fig 1). The number of beach patches is determined by the parameter *beach-density*, i.e. the fraction of beach patches, while the fraction (1- *beach-density*) of patches is equally divided between water and land. To create a spatial distribution closer to a real scenario, instead of dividing the landscape into simply randomly chosen beach, land and water patches, we created processes to scatter the land and beach patches over the water landscape. After scattering them, we classified the non-water patches into two categories: the land (the patches surrounding the starting point of the scattering process) and the beach (the patches further away).

The model is characterised by a set of state variables: the study parameters, the agents’ variables and the global variables. The study parameters (Table 1) are defined by the user in each simulation as a configuration of an experiment, determining a scenario and remaining constant during a simulation run.

**Table 1:** Study parameters.

Parameter name	Brief description
*beach-density*	Percentage of beach patches of the total number of patches in the environment.
*people-density*	Number of people compared with the total number of patches.
*beached-whale-distribution*	Type of beached whale distribution in the space, i.e. uniform (every beach patch has the same probability of beaching) or gaussian-σ (the beaching probabilities of beach patches follows a 2D Gaussian with the mean placed at the middle of the space and a standard deviationσ that modulates the spatial dispersion of beachings).
*prob-beached-whale*	At each time step, a whale beaches with a probability *prob-beached-whale*.
*movement*	Type of people agents’ movement, i.e. random walk or Lévy flight (modelled as a truncated Cauchy function)
*distance-walked-per-tick*	In random walk movement, it is the distance (measured by the the number of patches) that a people agent can walk each time step.
*vision*	A whale can be seen within a maximum circular area of radius *vision*. This parameter is measured by the number of patches.
*signal-range*	Maximum distance that a signal (e.g. smoke) created by people to announce a beached whale can be seen. It is measured by the number of patches.
*prob-mutation*	Probability of an error or an exploratory strategy in the imitation of people’s strategies.
*rounds-per-generation*	People can change their decision strategies every *rounds-per-generation* time steps.
*social-capital-vs-meat-sensitivity*	Relative importance of the social capital compared with meat in the fitness function of a people agent (Eq 4). This ranges from 0 to 1, where 1 implies that the meat has no importance for the fitness of a people agent.

People and whale agents are characterised by the state variables that appear in Table 2 and Table 3, which can be changed during a simulation. The global parameters are accessory parameters that complete the definition of the model (Table 4).

**Table 2:** People state variables.

Parameter name	Brief description
*prob-cooperation*	A people agent that has found a (non-public) beached whale makes it public, i.e. makes it much more visible, with a probability *prob-cooperation*.
*last-public-prob-cooperation*	This variable is updated with a people agent’s *prob-cooperation* if she has found a whale and any other people agent has seen her making it public (cooperating) or doing the opposite (defecting).
*meat*	Stock of whale meat held by a people agent (Eq 2 and Eq 5).
*social-capital*	Stock of social capital acquired by a people agent (Eq 3 and Eq 5).
*fitness*	Success of a people agent’s strategy, which is determined by the variables *meat* and *social-capital* (Eq 4).
*reputation*	Reputation of a people agent. This ranges from 0 to 1 (Eq 1)
*n-calls-history*	Vector of size *history-size* that contains the number of times a people agent has been seen cooperating in the last *history-size* periods.
*n-been-caught-history*	Vector of size *history-size* that contains the number of times a people agent has been seen defecting in the last *history-size* periods.

**Table 3:** Whale state variables.

Parameter name	Brief description
*my-range*	Radius of visibility of a whale (measured by the number of patches). This radius is equal to *vision* (people’s vision) if the whale is not public, and equal to *signal-range* of the whale has been made public.
*caller*	People that made the whale public by making a call.
*public*?	Boolean. True if the whale is public, false otherwise.
*life*	Number of periods the whale remains in the environment until it has completely rotted.

**Table 4:** Global parameters.

Parameter name	Brief description
*beach-whale-life*	Percentage of the *rounds-per-generation* that a beached whale stays in the model. It is set to 0.5.
*history-size*	Size of the vectors *n-calls-history* and *n-been-caught-history*
*history-past-discount*	The discount factor *δ* of Eq 1 that takes into account how important the shadow of the past is in terms of reputation.
*marginal-function-alpha*	Term *α* of Eq 2 and Eq 3. It is set to 0.1.
*marginal-function-mu*	Term μ of Eq 2 and Eq 3. It is set to 0.
*cauchy-scale*	Scale parameter of the truncated Cauchy distribution for Lévy flight movement, which specifies the half-width at half-maximum. It is set to a value such that the average agents’ step length per tick corresponds to one of the study values {4,6,8}.
*cauchy-location*	Location parameter of the truncated Cauchy distribution for Lévy flight movement, specifying the location of the peak of the distribution. It is set to 0.
*gaussian-std-dev*	Standard deviation σ of the Gaussian *beached-whale-distribution* determined by the type of *gaussian-σ*.

The environment is a 2D space whose dimensions can be adjusted to accommodate different spatial scales. The temporal scale of the model is also flexible. Each simulation time step (i.e. tick) can vary from days to weeks or months, depending on the values of the parameters: *prob-beached-whale*, which determines the number of whales beached in a period; and *distance-walked-per-tick*, *cauchy-scale* and *cauchy-location*, which define the distance people move each tick depending on the type of movement.

#### Overview: process overview and scheduling

**Fig 2:**
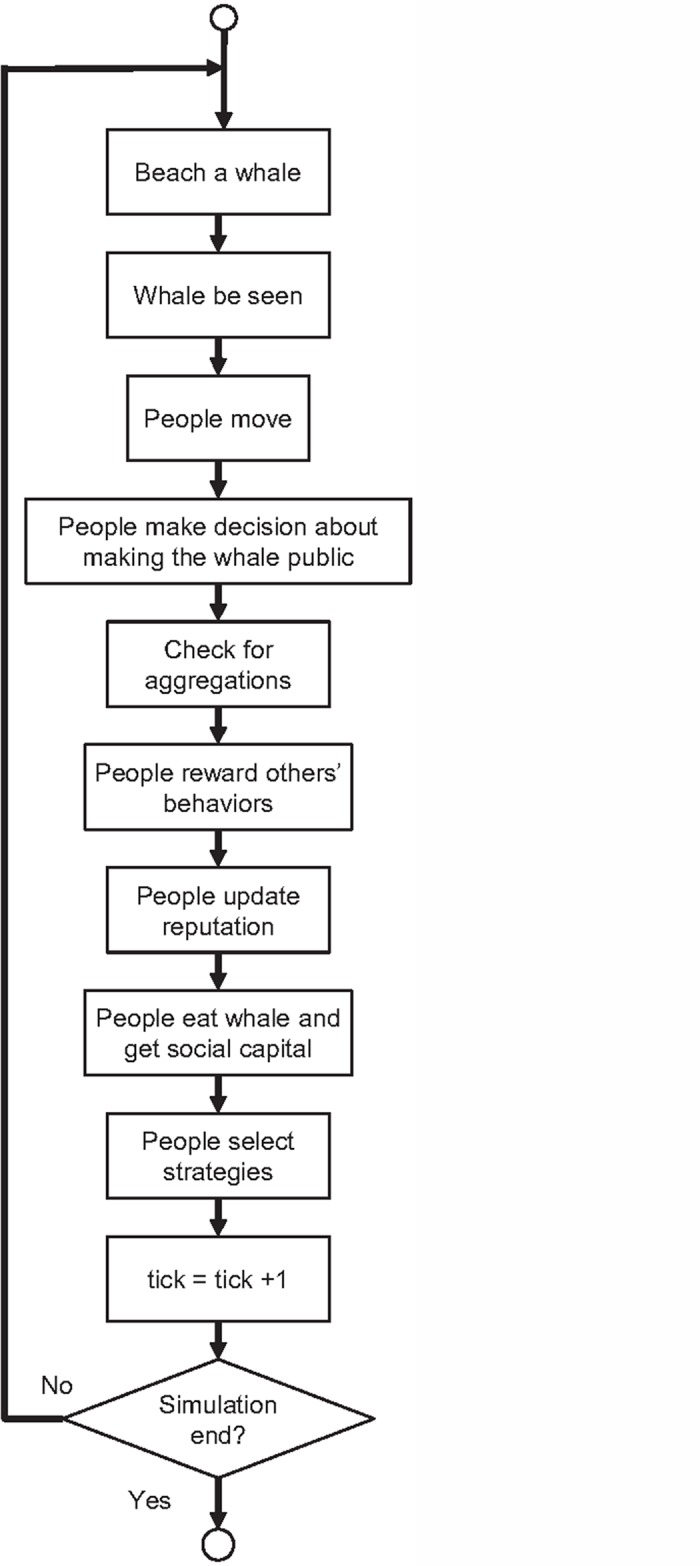
Diagram of the implementation schedule.

The scheduling of the model execution in discrete time steps is shown in Fig 2. The submodels are explained in detail in the Submodels subsection. The order in which the agents perform the actions is random, avoiding privileging first-acting consequences. The update of the state variables is asynchronous.

#### Design concepts: basic principles

The basic principle underlying this model is the cooperative phenomenon of indirect reciprocity. When Yamana people find a beached whale, they face the dilemma of whether to cooperate and share the resource with other Yamana or to defect and exploit the whale alone. They have established a reputation mechanism to promote cooperation and punish defections. Moreover, the model incorporates the basic principle of an evolutionary mechanism in imitating the individual strategies.

#### Design concepts: emergence

This model shows the emergence of cooperation or defection in Yamana society, a non-trivial outcome that depends on the characteristics of beachings (e.g. stochasticity, spatial distribution, visibility), human walking patterns (e.g. Lévy fligt, random walk), and other social features such as the reputation and imitation processes.

#### Design concepts: adaptation

The only adaptive trait of people agents is the cooperative trait, i.e. *prob-cooperation*, which determines the probability of cooperating when a people agent finds a beached whale.

#### Design concepts: objectives

The success of people agents is measured by the fitness function (Eq 4), regarding the incomes of meat and social capital people achieve, which correspond respectively to food and social resources. Social capital abstracts benefits that contribute positively to the agent’s fitness generated from diverse social activities such as knowledge transmission, cultural interchange or social relationships, in contrast to pure eating resources collected by food variable. The relative importance of meat versus social capital is weighed by the parameter *social-capital-vs-meat-sensitivity*.

#### Design concepts: learning

The model reproduces an evolutionary mechanism of imitation of successful strategies.

#### Design concepts: sensing

People can perceive the presence of whales in their surroundings, limited by a certain vision range.

#### Design concepts: interaction

When an aggregation occurs, i.e. a whale is found, people can interact with others by means of: rewarding cooperation and punishing defection (reputation Eq 1), and sharing meat and social capital.

#### Design concepts: stochasticity

The submodels that include stochasticity are: the beaching of whales, the patterns of movement (random walk and Lévy flight), the probability of cooperating (sharing a beached whale), the imitation of strategies and the probability of mutation.

#### Design concepts: collectives

When two or more people are in the location where a whale beached, an aggregation event occurs, with activities of reward and punishment for behaviours (reputation), sharing meat and exchanging social capital.

#### Design concepts: observation

**Fig 3:**
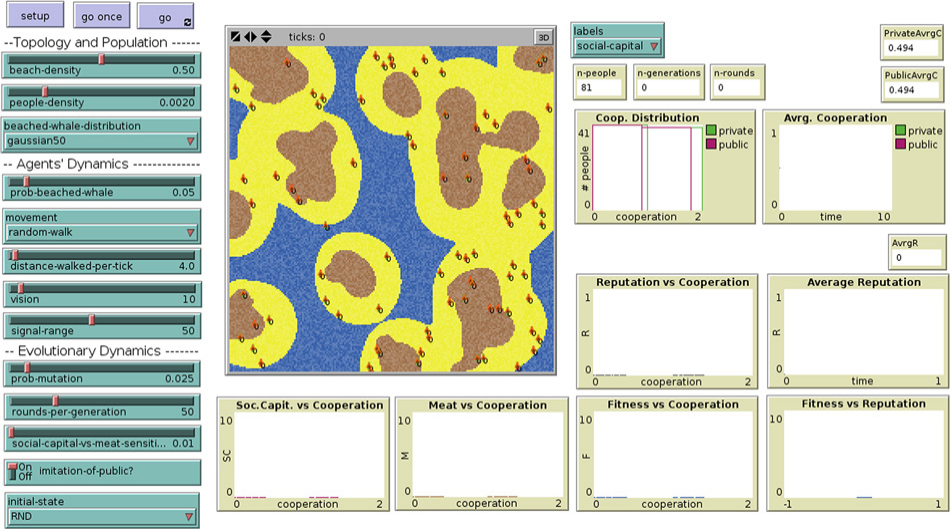
Interface of the model implementation in NetLogo.

Observation of the model includes the spatial distribution of people and whale agents, the average probability of cooperation, the average reputation of people and other social and economic magnitudes as seen in Fig 3.

#### Initialization

The user initializes the process by selecting the study parameters’ values in the interface, corresponding to the scenario to be simulated. The agents and the environment are then created according to this parameterisation.

#### Submodels

The model consists of the following submodels (Fig 2) that are executed sequentially.

#### Submodels: beach a whale

Each tick, a whale beaches with a probability *prob-beached-whale*. The beaching place is selected between beach patches according to the *beached-whale-distribution*, i.e. uniform (every beach patch has the same probability of beaching) or *gaussian-σ* (the beaching probabilities follow a 2D Gaussian with mean placed at the middle of the space and a standard deviation σ that modulates the spatial dispersion of beachings).

#### Submodels: whale be seen

Whales can be seen within a circular area defined by a radius *my-range* (of each whale agent). People agents inside this circular area that were not heading toward another whale set this whale as their target and move toward this whale in the following ticks.

#### Submodels: people move

If a people agent has seen a whale (i.e. her target), she walks towards the target whale the average distance for the type of walk pattern. Otherwise, she moves according to the walk pattern selected for the simulation. For the random walk pattern, the people agent walks a distance *distance-walked-per-tick* in a random direction. For the Lévy flight pattern, the direction of the movement is also random, but the distribution of steps lengths has been modelled with a Cauchy distribution, which is a particular case of the Lévy or stable distributions with a characteristic exponent α = 1 and that corresponds to the optimum exponent μ = 2 of the Lévy foraging hypothesis [33,56]. In our implementation, like in Viswanathan’s for Lévy flight [33] [33], when a walker sees a target, the step length is truncated; this is sometimes called “target-truncated Lévy Flight” but it is nothing more than the standard Lévy flight [56]. In addition, we have implemented the truncated Lévy flight [33,57], in which the tails of the probability distribution are limited in the upper side to avoid the physical impossibility of a distribution of step lengths with diverging variance in nature. Hence, the distance a people agent walks per tick follows a truncated Cauchy distribution of parameters *cauchy-scale* and *cauchy-location*, with a minimum distance of 1, corresponding to a movement of one patch distance, and a maximum equal to the half of the side of the 2D square world.

#### Submodels: people make a decision about making the whale public

A people agent that has found a whale makes the decision whether to call other people (cooperate) and share the whale, or to keep it for herself (defect). A people agent cooperates with a probability *prob-cooperation* (agent’s variable). If the people agent decides to make a call to announce the beached whale, the whale becomes public (whale’s state variable *public*? is set to true) and the range within which the whale can be seen is incremented from *vision* (natural distance in which a people agent can see a food resource) to *signal-range* (range of call signals).

#### Submodels: check for aggregations

A whale in whose location there are two or more people agents is considered an aggregation.

#### Submodels: people reward others’ behaviours

People’s reputation is a function (Eq 1) of their individual history of past actions. This history keeps the last *history-size* generations of two vectors: *n-calls-history* and *n-been-caught-history*. If a people agent decides to call everyone else (cooperate) when she finds a (non-public) beached whale, and there is at least a witness to her action, she adds a unit in the current generation period of *n-calls-history* (once per aggregation). However, if the agent decides not to call others (defect) and there are witnesses to that defection, she adds a unit (per beach not shared) in the current generation period of *n-been-caught-history*.

#### Submodels: people update reputation

Each people agent updates her reputation *R*_*i*_ computing the division of two moving averages:

$$R_{i}=\;\frac{\sum_{j=1}^{h}\left(\#Cooperate_{j}\right)\delta^{j}}{\sum_{j=1}^{h}\left(\#Cooperate_{j}\cup \#{\text{BeSeenDefecting}}_{j}\right)\delta^{j}}\in \left[0,1\right]$$ Eq.1

Where *#Cooperate*_*j*_ is the *jth* element of the vector *n-calls-history* and *#BeSeenDefecting*_*j*_ is the *jth* element of the vector *n-been-caught-history*. The δ ∈ [0,1] is the global parameter *history-past-discount*, which takes into account the shadow of the past, i.e. the importance of past events in the present. A value close to 1 gives the same importance (the maximum) to the past as to the present; a value close to 0 despises past events.

It is important to note that the reputation of a people agent is only affected by cooperation or defection actions conducted while been observed by others. If she defects but is not caught, her reputation does not change; similarly, if she cooperates but nobody answers the call, her reputation does not change either. This feature matches the hypothesis that reputation is a kind of social tag that someone always receives from others and cannot be changed by the owner. In the particular case that a people agent does not have history of past cooperation and/or defection actions, she takes the current average reputation of the population as hers.

#### Submodels: people eat whale and get social capital

We model a stylised abstraction of the process of getting *meat* and *social-capital* (people’s variables) assuming that the number of ticks that a whale stays in the model is fixed (*beach-whale-life* global parameter), and the marginal gain (gain per tick) of these stock variables for any individual depends only on their reputation and the number of people in the aggregation at each moment. In particular, the marginal gain of meat a people agent can achieve ΔM_i_(t) depends on the number N of people in the aggregation as follows:

$$\Delta M_{i}\left(t\right)=e^{-\alpha {\left(\left(N\left(t\right)-1\right)-\mu \right)}^{2}}\;with\;\;\Delta M_{i}\left(t\right)\geq 0$$ Eq.2

This equation formalizes a bell curve of parameters *α* and *μ*. The function shows increasing and decreasing returns depending on the value of *μ*. In order to represent the most critical scenario for the evolution of cooperation, *μ* is set equal to zero so that we focus our study in the part of the function with decreasing returns.

Besides, in an aggregation event, people exchange social capital. We suppose that the amount of social capital that a people agent can get is modulated by her reputation (a people agent with a bad reputation is not desirable company in an aggregation), and increases with the number of people in the event. The marginal social capital gain per tick ΔSC_i_(t) that a people agent can get in an aggregation of size N is described with the equation:

$$\Delta SC_{i}\left(t\right)=R_{i}\left(1-e^{-\alpha {\left(N\left(t\right)-1\right)}^{2}}\right)\;with\;\;\Delta SC_{i}\left(t\right)\geq 0$$ Eq.3

Where *α* is the same parameter as in Eq 2.

This function monotonically increases with the number of people N in an aggregation, and has a higher asymptote at the reputation of the agent *R*_*i*_. This behaviour fits with the hypothesis that the social capital increases with the number of people until a maximum, in which new people suppose redundant information or a limitation in the exchange of social capital. S1 Fig shows the plots of the curves of the marginal gain of meat and social capital (Eqs 2 and 3).

#### Submodels: people select strategies

The success of a people agent’s strategy is quantified with the fitness function:

$$F_{i}\left(t\right)=\theta SC_{i}\left(t\right)+\left(1-\theta \right)M_{i}\left(t\right)\;\;with\;\;\theta \in \left[0,1\right]$$ Eq.4

where

$$\begin{array}{l} SC_{i}\left(t\right)=SC_{i}\left(t-1\right)+\Delta SC_{i}\left(t\right)\\ M_{i}\left(t\right)=M_{i}\left(t-1\right)+\Delta M_{i}\left(t\right) \end{array}$$ Eq.5

The fitness function is weighted by a parameter *θ* that represents the relative importance of social capital over meat, i.e. *social-capital-vs-meat-sensitivity* parameter, allowing us to explore how this relative importance affects the evolution of cooperation.

Every generation, i.e. a period of *rounds-per-generation* ticks, people can imitate the best strategies of other people. The process of strategy imitation is similar to a roulette wheel, where each people agent randomly chooses another from the population with a probability directly proportionate to fitness; if the picker has less fitness, she copies the *last-public-prob-cooperation* of her choice. The hypothesis behind this assumption is that one can only imitate the observable behaviour of people. Each people agent updates her *last-public-prob-cooperation* variable with her *prob-cooperation* value whenever her behaviour is made public, i.e. she finds a beached whale, she calls other people and someone answers the call, or she does not call and someone sees the defection.

In addition, there may be some errors in the imitation process or a people agent may deliberately decide to explore new strategies, so a people agent chooses randomly with a probability *prob-mutation* a strategy between the strategy space.

After this imitation process, the people’s state variables meat, social capital and fitness are initialised to zero, while the reputation and past history vectors keep their values.

### Computational analysis techniques

In order to study the general behaviour of the model and the interactions between the model parameters and the output dynamics, we have applied Latin Hypercube Sampling, Classification and Regression Trees and Random Forests.

Exploring parameter space in ABM is generally difficult when the number of parameters is quite large. There is no a priori rule to identify which parameters are more important and their ranges of values. Latin Hypercube Sampling (LHS) is a statistical technique for sampling a multidimensional distribution that can be used for the design of experiments to fully explore a model parameter space providing a parameter sample as even as possible [58]. It consists of dividing the parameter space into S subspaces, dividing the range of each parameter into N strata of equal probability and sampling once from each subspace. If the system behaviour is dominated by a few parameter strata, LHS guarantees that all of them will be presented in the random sampling.

The multidimensional distribution resulting from LHS has got many variables (model parameters), so it is very difficult to model beforehand all the possible interactions between variables as a linear function of regressors. Instead of classical regression models, we have used other statistical techniques. Classification and Regression Trees (CART) are non-parametric models used for classification and regression [59]. A CART is a hierarchical structure of nodes and links that has many advantages: it is relatively smooth to interpret, robust and invariant to monotonic transformations. We have used CART to clarify the relations between parameters and to understand how the parameter space is divided in order to explain the dynamics of the model. One of the main disadvantages of CART is that it suffers from high variance (a tendency to overfit). Besides, the interpretability of the tree may be rough if the tree is very large, even if it is pruned.

An approach to reduce variance problems in low-bias methods such as trees is the Random Forest, which is based on bootstrap aggregation [60]. We have used Random Forests to determine the relative importance of the model parameters. A Random Forest is constructed by fitting N trees, each from a sampling with dataset replacement, and using only a subset of the parameters for the fit. The trees are aggregated together in a strong predictor by means of the mean of the predictions of the trees that form the forest in the regression problem. Approximately one third of the data is not used in the construction of the tree in the bootstrapping sampling and is known as “Out-Of Bag” (OOB) data. This OOB data may be used to determine the relative importance of each variable in predicting the output. Each variable is permuted at random for each OOB set and the performance of the Random Forest prediction is computed using the Mean Standard Error (MSE). The importance of each variable is the increase in MSE after permutation. The ranking and relative importance obtained is robust, even with a low number of trees [61].

We use CART and Random Forest techniques over simulation data from a LHS to take an initial approach to system behaviour that enables the design of more comprehensive experiments with which to study the logical implications of the main hypothesis of the model.

## Results

### General behaviour

The parameter space is defined by the study parameters (Table 1) and the global parameters (Table 4). Considering the objective of this work, two parameters, i.e. the location parameter of the truncated Cauchy distribution *cauchy-location* and the peak location of the marginal gain of meat *marginal-function-mu*, have been removed of the LHS; for the remaining 18 parameters we have explored a range of values (Table 5) according to the characteristics of the case study, e.g. small dense population, medium beach density. Note that two of the parameters are discrete, i.e. *movement* ⊂{“random-walk”,”levy-flight”} and *beached-whale-distribution* ⊂{“uniform”,”gaussian”}, while the rest are continuous.

**Table 5:** Parameters of the LHS.

Parameters	Range explored
*beached-whale-distribution*	{*uniform;Gaussian*}
*movement*	{*random-walk;levy-flight*}
*beach-density*	[0.25,0.75]
*people-density*	[0.001,0.01]
*prob-beached-whale*	[0.01,0.5]
*distance-walked-per-tick*	[1,13]
*vision*	[2,50]
*signal-range*	[50,100]
*prob-mutation*	[0.01,0.1]
*rounds-per-generation*	[25,75]
*social-capital-vs-meat-sensitivity*	[0,1]
*beached-whale-life*	[0.25,0.75]
*history-size*	[1,20]
*history-past-discount*	[0.5,1]
*marginal-function-alpha*	[-1,0]
*cauchy-scale*	[1,5]
*gaussian-std-dev*	[5,100]

In order to carry out a LHS, we have divided the range of each continuous parameter into N = 4000 strata, compounded 4xN experiments (corresponding to product space of the two discrete parameters) in which each continuous parameter has been sampled randomly from one of its stratum randomly selected, and run each experiment 10^5^ time periods (i.e. time limit). For all simulations, the average cooperation, i.e. the average number of cooperators in the population, has been recorded.

We focus the analysis on the stationary regime of the system, at which the influence of the initial conditions has disappeared and the system state persists over time. The standard deviation of the average cooperation in the last 10,000 time steps of a run is very small for most of the experiments (S2 Fig), which is consistent with the assumption of a persistent regime at the previously fixed time limit.

**Fig 4:**
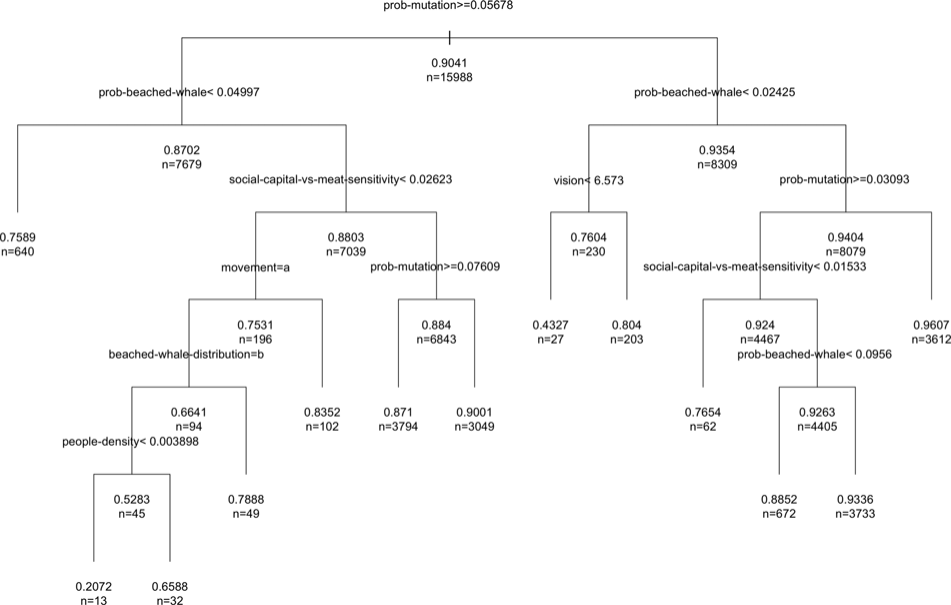
Pruned regression tree for average cooperation within the time limit. The CART uses the LHS data. Each decision node shows the condition used to divide the data, along with the number of runs after the split and the corresponding average of cooperation. The resulting subset on the left side satisfies the conditions while the subset on the right side does not. The maximum CART has been pruned with minsplit = 20 (i.e. the minimum number of observations that must exist in a node to try a split) and cp = 0.01 (i.e. complexity parameter).

A CART has been fit to the LHS data in order to enlighten the relationship between model parameters and the stationary behaviour as much as possible. The R package “rpart” [62] has been used to grow the CART tree until each node contains a small number of instances and then use cost-complexity pruning to remove irrelevant leaves. The resulting tree (after pruning) is too large to be easily understood since all parameters are important to a greater or lesser extent, so we have pruned the tree to improve interpretability using the parameters *minsplit* = 20 and *cp* = 0.01. The resulting pruned CART is showed in Fig 4.

Interpretation of the pruned tree should be prudent, because CARTs often show high variance (i.e. tendency to overfit the data). Therefore, the CART of Fig 4 is used as a first approach to system behaviour and a guideline to proceed with a more exhaustive computational analysis described in the next section. The first decision node in the CART uses the *prob-mutation* parameter in the condition. The particular condition value can be interpreted as a split point between exploration and learning of strategies. Small values of mutation limit the probabilities of escape for those states determined by the main forces that lead agents’ learning, i.e. the indirect reciprocity mechanism, the visibility and the stochasticity of beachings. For that reason, the detailed analysis of the model focuses on the first right leave of the CART.

**Fig 5:**
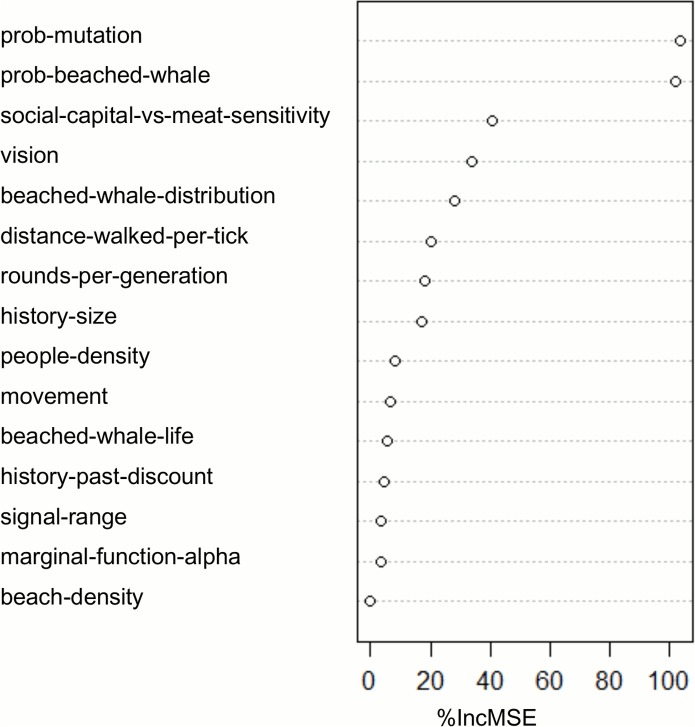
Parameter importance. A random forest with mtry = 18/3 (where 18 is the number of parameters) and ntree = 300 (for this value the MSE is stabilised) has been implemented. The permutation-based MSE reduction is used as the criterion of importance to rank the model parameters. By randomly permuting predictors (i.e. parameters) and observing how much the MSE grows, the more important a predictor, the more increase in the MSE is expected.

To solve the overfitting problem and to get a better understanding of the model parameters, we have used a Random Forests implemented with the “randomForest” R package [61]. Fig 5 shows the parameter importance using the Mean Standard Error (MSE) reduction of each permuted parameter over the OOB dataset [60]. The interpretation of these results is much more trustworthy because these importance predictions with a Random Forests are more stable and robust to changes in data [61]. The results confirm the importance of the mutation parameter along with *prob-beached-whale*, *social-capital-versus-meat-sensitivity*, *vision*, *beached-whale-distribution* and *distance-walked-per-tick* (all of them with over 20% increase in the MSE), which govern the main hypothesis of the model, from indirect reciprocity to the beachings and agents’ movement.

### Comprehensive design of experiments

Once the model has been analysed to understand the relative importance of the parameters in terms of the level of cooperation reached in the population, we focus the analysis on the two fundamental aspects of this article: the type of movement and the spatial correlation of the resource distribution. More specifically, we have carried out a set of simulations in order to explore the influence of the: (1) different importance levels of indirect reciprocity (*social-capital-vs-meat-sensitivity* (θ) parameter), (2) different probability spatial distributions of beaching events (*beached-whale-distribution* parameter) and (3) different types of movement of the agents on the space (*movement* parameter). In all scenarios, we also test the influence of the frequency of beaching (*prob-beached-whale* (P_bw_) parameter) and its visibility (*vision* (v) parameter). Table 6 shows the set of parameters that defines an experiment; the rest of the parameterisation is described in S1 Table.

**Table 6:** Comprehensive design of experiments.

Parameters	Symbol	Values explored
*social-capital-vs-meat-sensitivity*	*θ*	*θ ∈* {0,10^−2^,10^−1.5^,10^−1^,10^–0.5^,1}
*beached-whale-distribution*		{*Uniform;Gaussians*(20,40,80)}
*movement*		{*random-walk;levy-flight*(4,6,8)}
*prob-beached-whale*	*P*_*bw*_	*P*_*bw*_ *∈* {0.05,0.2}
*vision*	*v*	*v ∈* {5,10,20,30,40}

Each experiment has been replicated 30 times. The maximum standard error for the statistics is included in the legends of the corresponding figures.

The ultimate goal is to better understand the behaviour of the model when the learning process (the selection of the strategies with higher success) dominates the dynamics, so the mutation level chosen has been fixed at a relatively high level in such a way that exploration of new strategies is still present in the model, but not so much as to break this dominance. We have replicated several random and independent samples for each experiment to get statistics accurately enough. In short, the purpose is to study to what extent this combination of factors promotes or does not cooperation.

### Indirect reciprocity and cooperation

The WWHW model implements an indirect reciprocity mechanism that promotes cooperation, a stylised abstraction of the one used in Yamana society [12]. As we described in the Methods section, this social mechanism is based on a reputation variable that relates the public history of an individual agent and determines her capacity to gain social capital from others when she participates in an aggregation. The *social-capital-versus-meat-sensitivity* parameter (*θ*) modulates the relative importance of social capital in the agents’ fitness function, and consequently the efficacy of the indirect reciprocity mechanism to promote cooperation, i.e. the more importance of social capital, the more influence of reputation on agents’ fitness.

**Fig 6:**
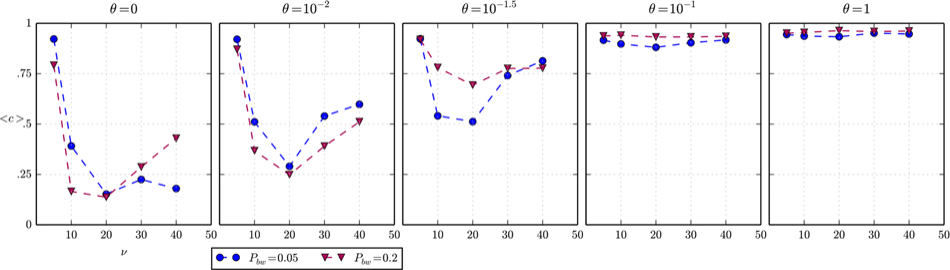
Average cooperation and importance of social capital. Row of plots of the average cooperation *<c>* as a function of vision *v* for different levels of importance of social capital *θ*, when the spatial distribution of beached whales is uniform in the space and the agents’ movement is a random walk. The maximum standard error of the average of cooperation of all experiments represented in the plots is 0.056.

Fig 6 shows the results of a set of experiments carried out to study reciprocity under different scenarios of the frequency of beachings (*P*_*bw*_)and the visibility of these events (*v*). An exhaustive analysis is described in Briz et al [12]. In this paper we replicate these results and summarise those that appear to be most relevant for the rest of this work.

When the indirect reciprocity mechanism does not have an effect on the system behaviour (θ = 0), which corresponds to a limiting case of our society of hunter-fisher-gatherers when the agents’ fitness is driven only by the consumption of meat, the level of cooperation is low in almost all values of vision, with the exception of a quite low value (*v* = 5). By contrast, cooperation grows with the importance of social capital *θ* and consequently the indirect reciprocity mechanism gains influence, approaching close to 1 for values of *θ*>0.1. These results are to be expected, considering the nature of the social mechanism of indirect reciprocity implemented in the model. It is much more significant to observe the effect of the parameter *vision*; since *vision* determines the visibility of beached whales and the chances of detecting defectors, there is a trade-off that explains that for low vision values *v* = 10 there are more chances for a defector to consume whale alone and escape punishment; but greater values of vision increase the opportunities of having to share the whale and simultaneously being punished and acquiring a low reputation when defecting.

More interesting is the case of *v* = 5, where full cooperation is reached even for *θ* = 0. This counterintuitive result is due to the hypothesis of the WWHW model, which assumes that only public behaviours can be imitated. The cooperative strategy always becomes public because people come to the call of a cooperator, but a defection is rarely detected for low values of vision and is rarely made public as a result. Therefore, the selection process mainly operates under the cooperative strategy. In short, for low values of vision the model reproduces a case in which there is a public-private discrepancy in the imitation, i.e. people imitate more successful (private) strategies, but they also copy public information available about these strategies which may not correspond to the real (private) strategies. In fact, this happens at the early stages of the simulation, where there are defectors that are not being caught, hence their reputation is still good (cooperator-like).

### Spatial concentration of beachings and cooperation

**Fig 7:**
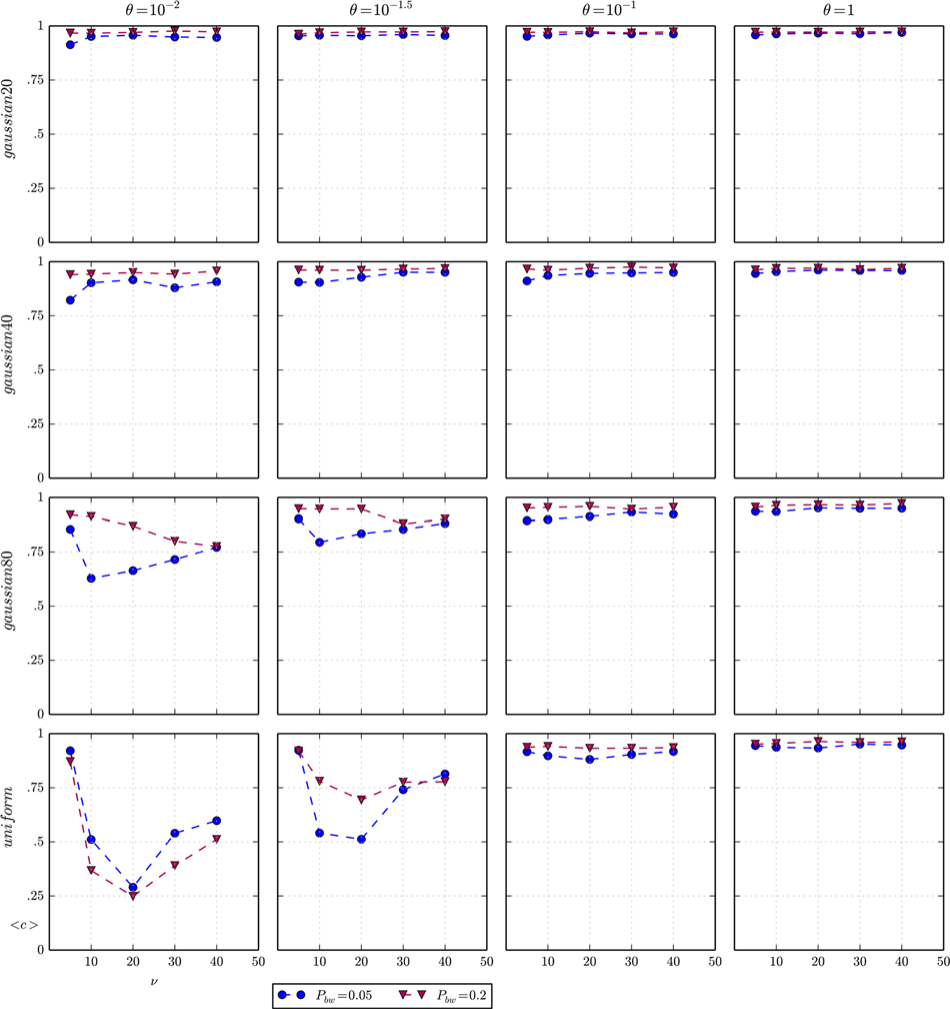
Average cooperation and spatial distribution of beached whales. Matrix of plots of the average cooperation *<c>* as a function of vision *v* for different spatial distributions of beached whales (columns) and levels of importance of social capital *θ* (rows), when the agents’ *movement* is a random walk. The maximum standard error of the average of cooperation of all experiments represented in the plots is 0.056.

In the next set of experiments, we relax the assumption that beached whales are uniformly distributed over the space and consider other families of distributions closer, or at least more plausible, to the historical distribution of beachings. In particular, we suppose that beached whales follow a 2D Gaussian with the mean placed at the middle of the space and a standard deviation σ that modulates the spatial dispersion of beachings. Fig 7 shows the level of cooperation for a combination of different spatial distributions, i.e. uniform and Gaussians, and levels of importance of social capital *θ*, when the frequency of beachings *P*_*bw*_ and the visibility of these events *v* vary. The bottom row of plots corresponding to a uniform distribution is identical to the results showed in Fig 6, and can be used as a benchmark for comparing the effects of the set of Gaussian distributions, with increasing standard deviation σ, whose results are depicted in each of the remaining rows of Fig 7.

The conclusion is quite evident: in all parameterisation scenarios, the spatial concentration of beachings (five first rows of Fig 7) pushes up cooperation from the original levels reached by effect of the indirect reciprocity mechanism (bottom row of Fig 7). These results corroborate the intuitions about the Yamana case study: namely the spatial concentration of beachings, defined in the model by the parameters σ and *P*_*bw*_ respectively, favour cooperation. The explanation is that the spatial and temporal interactions of agents increase, and although any of these events may conclude in cooperation or defection, the characteristics of cooperative behaviour facilitate the emergence of communities of cooperators that persist in time. In the WWHW model, a cooperator always calls everyone else, and consequently attracts people to the group; contrarily a defector never calls and consequently tends to separate from the group. The cooperative behaviour prompts an unexpected mechanism of positive assortment, i.e. the probability of interacting with a cooperator is greater for a cooperator than for a defector, which promotes cooperation.

These dynamic communities (they continuously join and separate over time at the rhythm of meetings around a beached whale) show another feature that favours cooperation. The spatial proximity between agents works as a vigilance network that makes it very difficult for a defector not to be caught and consequently makes defection very costly. This effect becomes much more important as the importance of social capital grows in the society (given any spatial distribution, note that the cooperation levels increases with *θ* in Fig 7).

The simulation results from the spatial distribution experiments we have just described, which show that communities of cooperators required for supporting cooperation do not need to be formal, i.e. agents know the community to which they belong perfectly; they may simply be a result of informal meetings that repeat over time in a specific area. Within these informal groups, two concurrent mechanisms seem to promote cooperation: the positive assortment of cooperators and the vigilance network.

### Lévy flight movement and cooperation

**Fig 8:**
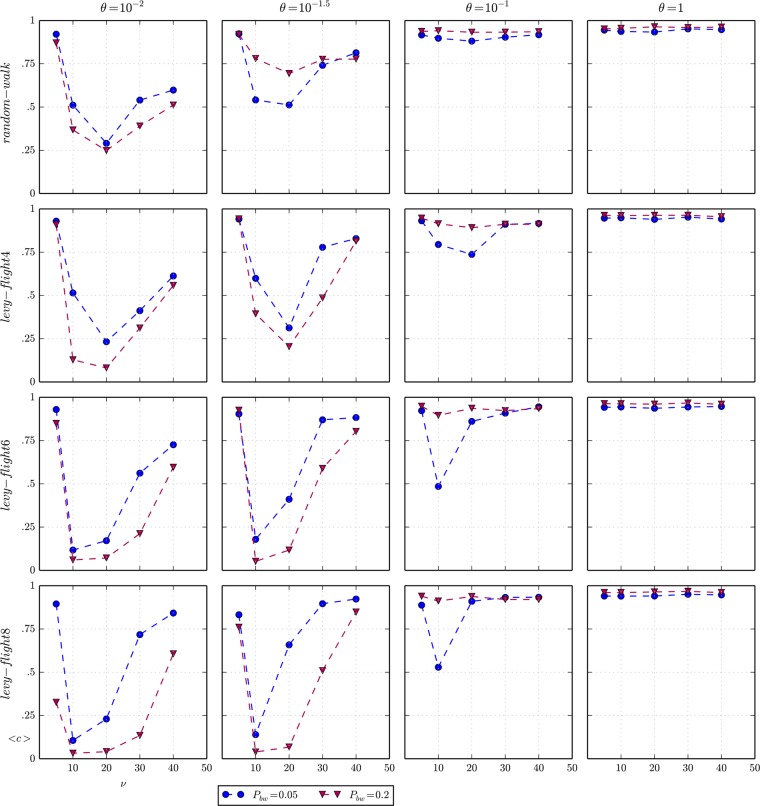
Average cooperation and movement. Matrix of plots of the average cooperation *<c>* as a function of vision *ν* for different agents’ types of *movement* (columns) and levels of importance of social capital *θ* (rows), when the spatial distribution of beached whales is uniform. The maximum standard error of the average of cooperation of all experiments represented in the plots is 0.056.

In the last set of experiments, we relaxed the assumption that agents move following a random walk. Now, we assume Lévy flight movement much more similar to real human mobility patterns discussed in the literature [31–33,35]. As we have just described in the Methods section, we have implemented a truncated Cauchy function for the agents’ step length per tick, with a minimum step length of 1, corresponding to a movement of one patch distance, and a maximum equal to the half of the side of the 2D square world. In order to compare this truncated power law distribution of step length with the original random walk of fixed step length of 4 (patches), we choose the Cauchy parameters such that the average length is fixed for a complete run. In particular we have explored a set of truncated Cauchy functions of {4, 6, 8} average step lengths whose results are shown in Fig 8. Now, the first row of plots corresponds to the random walk movement, identical to the results showed in Fig 6, and is used as a benchmark for comparing the effects of the increasing average step lengths of the Cauchy functions depicted in the remaining rows.

The average step length of an agent is directly related to her diffusion capacity, i.e. the distance at which an agent can interact with other agents and the environment. You could expect that greater diffusion capacity would cause the detection of “more things”, e.g. beached whales, defectors or callings by cooperators, because the effective seeking area would be broader to the extent that agents changed their seeking area more frequently, although its impact on the dynamics of the model may be more complex due to the *vision* parameter. Note that the type of movement determines the distribution of places (patches) reachable at each tick, while *vision* determines the seeking area from a place (patch) at each tick.

The effect of the Lévy flight movement is more visible for low values of *θ* ∈ {10^−2^,10^−1.5^} for which the indirect reciprocity mechanism is too weak and does not dominate the evolution of cooperation. An initial conclusion is that a “Lévy-flight4” movement with an average step length equal to the original random walk movement shows similar results than the last one (see the second row of plots of Fig 8), although the patterns of both movements are obviously quite different. Things change when the diffusive capacity of the Lévy flight movement increases.

**Fig 9:**
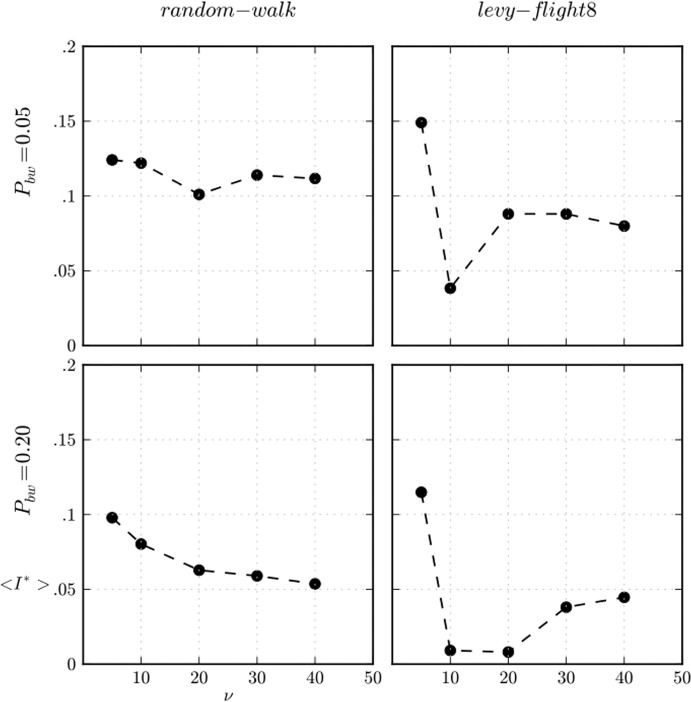
Public-private discrepancy in the imitations. Public-private discrepancy in the imitations for two values of *P*_*bw*_, and “random-walk” and “Lévy-flight8” movements, measured as the average imitations *I* ^*^ over a complete simulation run in which there was any discrepancy between the public and the private strategies of the agent imitated. The spatial distributions of beached whales is uniform and *θ = 10* ^*–1*.*5*^.

For low values of vision (*v* = 10), we see that the public-private discrepancy, which produced the emergence of unexpected cooperation when the movement was a random walk, decreases significantly (see the two first columns of plots in Fig 8). The higher diffusion capacity of Lévy flights {6, 8} increases the opportunities for detecting defectors, whose strategy now does take part in the selection process. To show this effect better, we have computed the average imitations *I* ^*^ in which there was any discrepancy between the public and private strategies of the agent imitated for a particular set of parameterisations (see Fig 9). Except of the limiting case *v* = 5 corresponding to high cooperation (see Fig 8), it seems clear that the more diffusive characteristic of Lévy flight reduces the discrepancy and consequently the abnormal levels of cooperation reached for low values of vision (*v* = 10).

**Fig 10:**
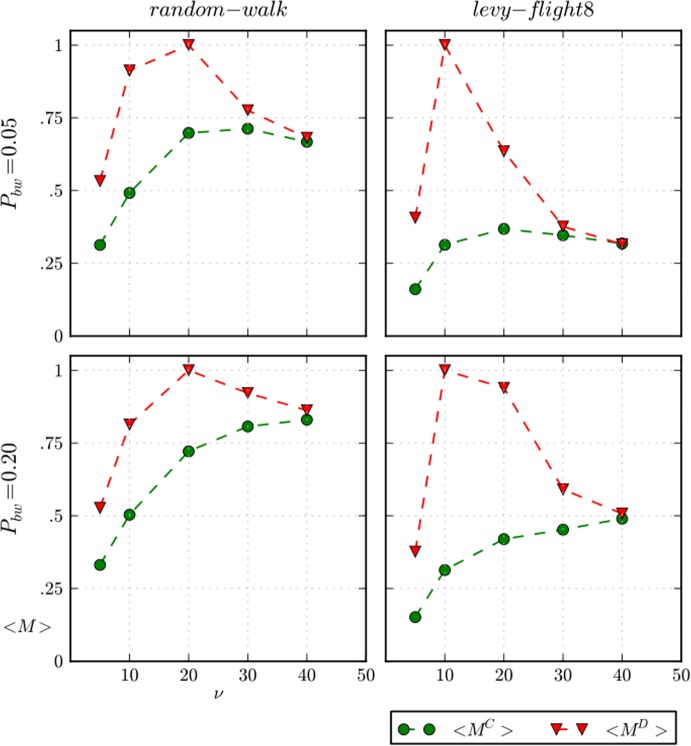
Average amount of meat for cooperators and defectors. Average amount of meat for cooperators *M* ^*C*^ and defectors *M* ^*D*^ over a complete simulation run for two values of *P*_*bw*_, and “random-walk” and “Lévy-flight8” movements. The spatial distributions of beached whales is uniform and *θ = 10* ^*–1*.*5*^.

Assuming Lévy flight movement, e.g. “Lévy-flight8” in Fig 8, the disappearance of the public-private discrepancy reveals the effects of vision on cooperation. When vision is low, a beached whale can be exploited almost exclusively, i.e. the probability of two or more agents finding a whale is quite low. This effect is readily observable in Fig 10, which depicts the average amount of meat *M* over a complete simulation run for defectors and cooperators. A defector gets high average *M* for low values of vision (*v* = 10), which is particularly significant with “Lévy-flight8” movement that reduces the public-private discrepancy. In contrast, a cooperator gets lower average meat *M* because she always calls and shares a beached whale, while a defector never calls.

**Fig 11:**
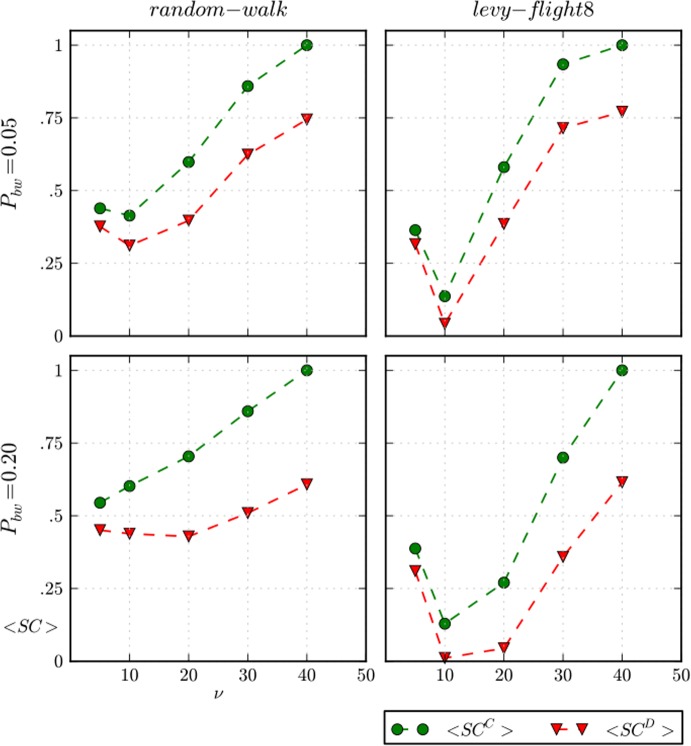
Average amount of social capital for cooperators and defectors. Average amount of social capital for cooperators *SC* ^*C*^ and defectors *SC* ^*D*^ over a complete simulation run for two values of *P*_*bw*_, and “random-walk” and “Lévy-flight8” movements. The spatial distributions of beached whales is uniform and *θ = 10* ^*–1*.*5*^.

However, the increase in the visibility of beached whales promotes cooperation to the detriment of defection. Fig 11 shows the average amount of social capital *SC* over a complete simulation run for defectors and cooperators, and is helpful for understanding this effect. Now, high values of vision *v* = {30,40}increase the probability of finding a beached whale for all people, as well as the chances that two or more agents might share the resource. Consequently, the average meat of defectors and cooperators converge as vision approaches the value of *signal-range* 50 (see Fig 10) because most agents share the food either intentionally (cooperators) or unintentionally (defectors). Simultaneously, whenever there is an aggregation, cooperators get social capital, as we can see in Fig 11, which results in higher fitness than defectors, who get lower social capital because they have lower reputations.

Finally, Fig 8 shows an interesting result. The effect of the Lévy flight movement is quite different depending on the frequency of beachings. Looking at the two first subplots of the case “Lévy-flight8” in Fig 8, particularly for intermediate and high values of vision (*v* ≥ 20), we observe that cooperation with *P*_*bw*_ = 0.05 is higher than with *P*_*bw*_ = 0.2. In both cases the higher diffusion capacity of Lévy flight reinforces the vigilance network, yet the cost of defection is more important in the case of scarce resources (*P*_*bw*_ = 0.05), pushing up cooperation. When the resource is more abundant (*P*_*bw*_ = 0.2) there are more opportunities for defectors to find a beached whale apart from the group of cooperators.

## Discussion

The results of the simulations reinforce the idea of preservation of the context, e.g. continuity of interaction patterns, as a key mechanism to foster and sustain cooperative behaviours in the analysis of social dilemmas [63,64]. Previous research has shown that spatial or structured contexts in the Prisoner’s Dilemma favour cooperation [65–70]. In fact, one of the proposed five rules for the emergence of cooperation [6], network reciprocity, is based on this effect. This behaviour is not exclusive to the Prisoner’s Dilemma, as the result has also been found in other games [71,72]. Nor can it be generalised for all social dilemma models [65,73] under all conditions [74–79].

Context preservation enhances the formation of communities based on frequent personal non-anonymous interaction, which typically increases the alignment of individual and collective objectives. This effect is sometimes referred to as *parochialism* [80,81]. Communities influence the formation of pro-social norms predisposing them to cooperation and attenuating coordination failures and the temptation of free-rider behaviour. The mechanism is usually explained by three forces [80]: 1) frequent interaction with the same agents stretches the shadow of the future [7] and consequently gives incentives to cooperate and promote reciprocity while avoiding punishing behaviour (*retaliation effect)*; 2) since the interaction takes place in a frequent and personal way and often with high clustering, low-cost access to information about the behaviour is available to the rest of the population—in fact, reputation, the basis for indirect reciprocity, requires that agents recognise and memorise others (*reputation effect)*; 3) In communities there is an additional tendency to encourage interactions among members of the same communities instead of having relations with outsiders. This shifts the balance in the pro-social direction as a consequence of interaction frequency [82] and is called the *segmentation effect*. Additionally, while it is possible to coordinate in stable unfair norms with members of other communities, it is more difficult among members of the same community [83–85].

The results of our model fit in the parochialism framework. Retaliation and reputation effects are driven by the indirect reciprocity mechanism of the model and exclusion in terms of social capital in case of an aggregation event, both based on the agent’s reputation. Although all the members included in the model are supposed to belong to the same community, in some sense the segmentation effect is also present as a consequence of the concentration of the population towards the resource when there is spatial correlation in the distribution of the resource. As a matter of fact, spatial correlation empowers all the mechanisms of parochialism. The roaming paths are close to the resource for all the agents, so the frequency of interaction and the possibility of gathering reliable information about the rest of the agents increase and the concentration of the population increases the odds of detecting a defector. The underlying network of vigilance is denser when the resource is not uniformly distributed, and higher clustering and interconnectedness promote cooperative social norms as previous research has indicated [86]. It is important to notice that this effect occurs even in this model in which the movement of the population is intentionally myopic. The agents are not endowed with memory about the location of the resource or learning capabilities to figure out the distribution. Higher levels of cooperation must be expected in populations that include any learning dynamics.

Applying these outcomes to Yamana society allows us to go beyond ethnographic accounts, which mainly emphasise the abundance of food provided by a cetacean stranding, yet several interesting implications may also be discussed. First, aggregation events engendered social networks, which in turn promoted cooperation and fuelled future events to the extent that they reinforced social norms and the sense of belonging, reducing information costs and allowing Yamana people to detect defectors. Once established, this network must have acquired its own dynamic enabling it to reproduce and maintain itself and become a constitutive part of Yamana society, shaping behaviours and practices. Thus, the self-identification of Yamana people to particular spaces, revealed by ethnographic data, could have been underpinned and enhanced by cooperative networks.

The higher frequency of aggregation events recorded in ethnographic sources, in comparison with the sparse data of defection, could be explained as a consequence of the payoff implied in cooperative networks in terms of socio-economical relationships. Although it is unclear when aggregations started between the hunter-gatherer societies under study, according to the dynamics set up in the model, it could be predicted that defection would decrease over time.

The ethnographical information about cooperative dynamics in Yamana society (within the context of an aggregation process) can be confirmed as reliable data with this model. At the same time, it allows us to explore the internal social mechanisms that establish and maintain social rules promoting cooperative attitudes. The ethnographic sources offer a general context of social rules of cooperation, not only at exceptional times of abundance, but also when sharing the products of everyday activities such as hunting, gathering and fishing.

From an archaeological point of view, the results of the simulations also allow a hypothesis about the spatial distribution of aggregation areas. The parochialism effect rose as a result of the heterogeneous occurrence of cetacean strandings, which would predict the potential areas where these events could have taken place.

Within our ethnoarchaeological framework, this point is especially relevant when trying to identify evidence of aggregation events in the archaeological record of the Beagle Channel [10,13,14]. Coastal sectors with higher probabilities of cetacean strandings must be prioritised in our quest of aggregation episodes to explore the archaeological markers of cooperation in Yamana society.

With regard to movement, previous research highlights the advantages of Lévy flight as a foraging strategy when target sites are sparse and can be visited recurrently [33,35]. These models align theory with the experimental foraging evidence found in different animal species and human communities. However, the influence that this type of movement may have on coupled social dynamics (e.g cooperative social norms) remains unknown. Results obtained with this model suggest that although movement patterns are not a key factor promoting or collapsing cooperation, Lévy flights can enhance cooperative behaviour modestly when the resource is scarce (defectors do not obtain the benefits of joint search) or/and the vision of agents is limited (unfolding the private strategies of the population).

Data provided by ethnographic sources suggest sporadic out-of-range movements between Yamana people (see above), including the effects of a “call” in case of whale stranding. Consequently, following the results of our model Lévy flight movement would have also involved a consistent mechanism of reinforcement of the cooperative rules in Yamana society that enables them to detect strandings and defectors.

## Conclusions

The Results section is mainly headed by two questions: how beaching spatial concentration and Lévy flight movement influence cooperation. The results have been discussed and addressed to the relevant literature in the Discussion section. They may be summarised as follows:

- The model presented here validates the dataset provided by historical sources in our Yamana case study. Our results highlight that cooperation practices (within the context of aggregation processes) were not an outcome of a fortuitous observation made by missioners or ethnographers in the early 20^th^ century. Conversely, cooperative behaviours were fuelled by a social dilemma and bolstered by a set of variables such us vision, reputation, mobility and stranding spatial distribution. This model allowed us to disentangle the mechanisms and conditions that promoted cooperation.
- The model presented here highlights that within the context of aggregation processes as documented in the ethnographic and historical sources, cooperation practices were fuelled by a social dilemma and bolstered by a set of variables such as vision, reputation, mobility and stranding spatial distribution. Conversely, the cooperative model allows us to disentangle the mechanisms and conditions that promote cooperation and enables us to transcend detailed and partial historical data.
- When beachings follow a 2D Gaussian, the spatial concentration of beached whales pushes up cooperation from the original levels reached by the effect of the indirect reciprocity mechanism. The cooperative behaviour favours the emergence and preservation of informal and dynamic communities that work as a vigilance network making defection very costly.
- When agents follow Lévy flight movement, assuming that a correlation exists between this movement type and the large average step length, the distance or effective vision at which an agent can interact with other agents and the environment grows, which means a greater ability to detect beached whales and more callings by cooperators and defectors. The ability to detect defectors removes the public-private discrepancy in the imitation of strategies that happens with random walks.
- Moreover, Lévy flight, with a large average step length, promotes cooperation when beachings are scarce. In this scenario, the higher effective vision extends the vigilance network discouraging defectors, who have few opportunities to prosper apart from the group of cooperators.
- If the assumed correlation between Lévy flight and the large average step length is absent, the movement pattern itself will not have as much influence in promoting cooperation, but the average step length of the movement will be enough to explain the phenomena.

## Supporting Information

S1 Fig Curves of marginal gain of meat (left graph) and social capital (right graph) corresponding to the equations Eq 2 and Eq 3 for different values of the parameter α. The parameter α in both equations governs how the marginal gain (per time step) of meat declines with the size of the aggregation N, and the marginal gain of social capital grows with the same aggregation size. We set α = 0.1 to make these decreasing and increasing returns consistent with the population scale determined by the parameter *people-density*.
(TIF)

S2 Fig Standard deviations histogram of the average cooperation in the last 10,000 time steps. For most of the runs of the LHS, the standard deviation is very small (the median is 0.0235), which is consistent with the assumption of a persistent regime reached by the system at the final time step of a run (set at 10^5^ time steps for all experiments).
(TIF)

S1 Table Parameterisation of the comprehensive design of experiments. (DOC)
