# Supplementary figures and images for: Effect of Resource Spatial Correlation and Hunter-Fisher-Gatherer Mobility on Social Cooperation in Tierra del Fuego
Source: PLoS One. 2015 Apr 8;10(4):e0121888. doi: 10.1371/journal.pone.0121888 (PMC4390331; doi:10.1371/journal.pone.0121888)

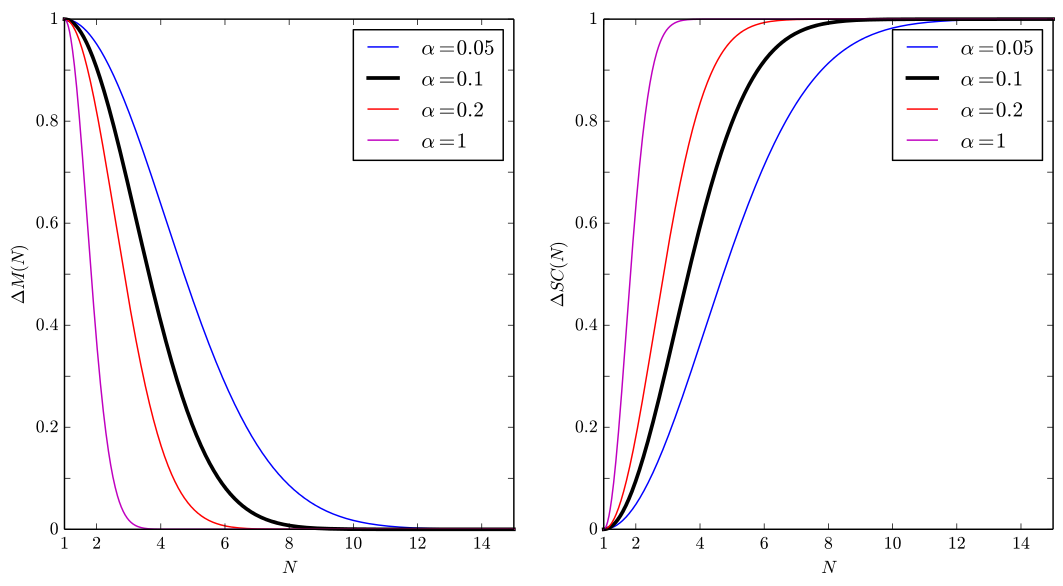

Supplement: S1 Fig — The parameter α in both equations governs how the marginal gain (per time step) of meat declines with the size of the aggregation N, and the marginal gain of social capital grows with the same aggregation size. We set α = 0.1 to make these decreasing and increasing returns consistent with the population scale determined by the parameter people-density. (TIF) [file pone.0121888.s001.tif]

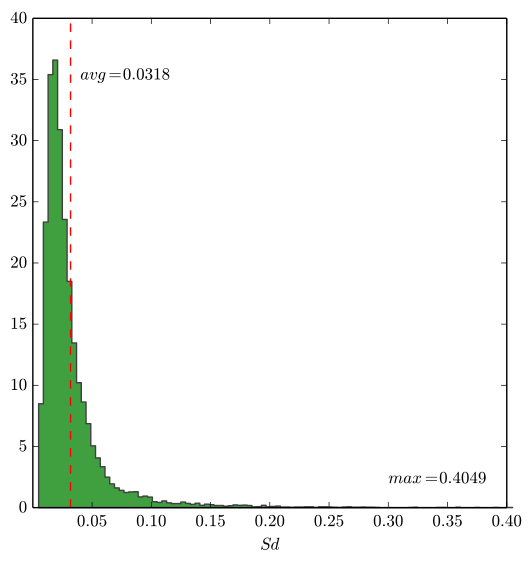

Supplement: S2 Fig — For most of the runs of the LHS, the standard deviation is very small (the median is 0.0235), which is consistent with the assumption of a persistent regime reached by the system at the final time step of a run (set at 105 time steps for all experiments). (TIF) [file pone.0121888.s002.tif]
