# Supplementary material for: Effect of Resource Spatial Correlation and Hunter-Fisher-Gatherer Mobility on Social Cooperation in Tierra del Fuego
Source: PLoS One. 2015 Apr 8;10(4):e0121888. doi: 10.1371/journal.pone.0121888 (PMC4390331; doi:10.1371/journal.pone.0121888)
Supplement: S1 Table — (DOC) [file pone.0121888.s003.doc]

| Parameter name | Value |
| --- | --- |
| *beach-density* | 0.5 |
| *people-density* | 0.0020* |
| *distance-walked-per-tick* | 4 |
| *signal-range* | 50 |
| *prob-mutation* | 0.025 |
| *rounds-per-generation* | 50 |
| *beached-whale-life* | 0.5 |
| *history-size* | 10 |
| *history-past-discount* | 0.8 |
| *marginal-function-alpha* | 0.1 |
| *marginal-function-mu* | 0 |
| *cauchy-location* | 0 |
| ** It corresponds to N=81 agents* |  |
